# The Nutrition Literacy Assessment Instrument for Brazilians, NLit-Br: An Exploratory Cross-Cultural Validity Study

**DOI:** 10.3390/nu14224914

**Published:** 2022-11-20

**Authors:** Lívia Botelho da Silva Sarkis, Juliana Teruel-Camargo, Heather D. Gibbs, Eduardo Y. Nakano, Verônica Cortez Ginani, Aline Silva de Aguiar, Camila dos Santos Chaves, Renata Puppin Zandonadi, Marcus Gomes Bastos

**Affiliations:** 1 School of Medicine and Health, Federal University of Juiz de Fora, Juiz de Fora 36038-330, MG, Brazil; 2 Department of Dietetics and Nutrition, University of Kansas Medical Center, Kansas City, KS 66160, USA; 3 National Heart, Lung, and Blood Institute, National Institutes of Health, 10 Center Dr/MSC 1825, Bethesda, MD 20892, USA; 4 Department of Statistics, University of Brasilia, Brasilia 70910-900, DF, Brazil; 5 Department of Nutrition, University of Brasilia, Brasilia 70910-900, DF, Brazil; 6 Department of Nutrition and Dietetics, Federal Fluminense University, Niterói 24220-900, RJ, Brazil

**Keywords:** cross-cultural adaptation, health literacy, nutrition education, Latin America, nutritional literacy

## Abstract

This study aimed to test the validity of the cross-cultural adapted Nutrition Literacy Assessment Instrument for Brazilians (NLit-Br). An observational cross-sectional study was performed in chronic disease clinics from the Brazilian Public Health System in two phases: (1) linguistic and cultural adaptation and (2) validity testing. Six registered dietitians and thirty adult patients diagnosed with at least one chronic disease participated in the study using the nutrition literacy assessment instrument (NLit-Br) and the short assessment of health literacy for Portuguese-speaking adults (SAHLPA-18). Sample descriptive variables: age, sex, race, income, education, and occupation. To adapt the instrument to the Brazilian Portuguese and Brazilian culture, we tested cognitive interviewing and the Scale Content Validity Index (S-CVI) with a group of dietitians and patients. To test the tool’s validity, health literacy (SAHLPA-18) was used as a construct that presents similarities and differences with nutrition literacy (NLit-Br). The correlation of NLit-Br and the SAHLPA-18 was tested (Spearman’s Rho). Internal consistency was measured by Kuder–Richardson Formula 20 (KR-20). The NLit-Br content validity (S-CVI = 0.85) and internal consistency (KR-20 = 0.868) were confirmed. Additionally, NLit-Br presented a significant and robust correlation with SAHLPA-18 (r = 0.665, *p* < 0.001). Therefore, the NLit-Br was considered a linguistic, cultural, and valid instrument to measure Brazilian’s nutrition literacy.

## 1. Introduction

A healthy diet is crucial for promoting and maintaining good overall health for all age groups. An unhealthy diet is a significant driver of non-transmissible chronic diseases (NTCDs), chronic inflammation, and adaptive immune system inhibition [1,2]. For decades, countries in Latin America have experienced a significant increase in obesity, diabetes, and cancer rates associated with rising physical inactivity and diets high in calories but low in nutrients [3,4,5]. As the largest and most populous country in Latin America, Brazil is deeply impacted by an unhealthy diet. Brazil ranks fourth in the world in cases of type 2 diabetes, and cancer is the second most common cause of death [6,7,8]. More recently, the country has struggled as the new epicenter of the SARS-COVID pandemic, with a highly vulnerable population due to the harmful impact an unhealthy diet has on the adaptive immune system [2].

Nutrition literacy (NL), or the individual ability and skills to understand, comprehend, and apply nutrition information, predicted healthier diet patterns in an adult population [9]. The concept of NL has been conceived using a similar theoretical framework as health literacy (HL) but incorporating the specificities that are singular to nutrition, e.g., interaction of food and health, food nutrient content, and food marketing [10,11]. Identifying NL levels can support targeted nutrition interventions to promote health, prevent NTCDs and improve the adaptive immune system.

Two instruments have been utilized to measure NL in Brazil: the Nutrition Literacy Scale (NLS) and the Newest Vital Sign (NVS) [12,13]. The NLS is a 28-item instrument that focuses on reading and comprehension of nutrition concepts and recommendations (e.g., calcium is essential for bone health, recommended fruits and vegetables portions per day, and others) [12]. The NVS is a 6-item tool that measures the food label interpretation of an ice cream. The instrument focuses on mathematical skills to calculate calories and nutrients in the food label, knowledge about the recommended intake of saturated fat and energy, and how to detect allergenic components in the product [13]. However, although both instruments target the crucial concepts of nutrition, both instruments need to include other essential concepts for healthy eating, such as knowledge of food groups, portion sizes, and the ability to navigate food marketing. Moreover, even though both instruments were translated into Brazilian Portuguese, none performed cultural adaptation based upon community-participatory research including both health professionals and communities to test the relevance of concepts, not only words, before applying the instrument in the general population.

Few studies were performed in Brazil using instruments to measure NL [12,13], but none using the Nutrition Literacy Assessment Instrument (NLit) that was developed to assess nutrition literacy in terms of nutrition knowledge and skills in making food choices [14]. The instrument has six subscales, with 64 items: (1) ‘Nutrition & Health’ which measures reading comprehension of the summarized Dietary Guidelines for Americans; (2) ‘Energy Sources in Food’ which measures knowledge of the macronutrient sources in food; (3) ‘Household Food Measurement’ which measures identification of recommended portions; (4) ‘Food Label and Numeracy’ which measures ability to apply information obtained from the nutrition facts panel; (5) ‘Food Groups’ which measures ability to classify foods by nutrition category, and (6) ‘Consumer Skills’ which measures ability to navigate food products to make healthy food choices. Currently, the NLit is validated and available in three languages: English [15], Spanish [16], and Italian [17]. For this study, we had two aims: (1) to adapt the NLit to Brazilian culture and Brazilian Portuguese (NLit-Br), and (2) to test the NLit-Br validity.

## 2. Materials and Methods

### 2.1. Study Design

This was an observational, cross-sectional study. All data were collected between August and November 2016. The University Research Ethics Board reviewed and approved the study protocol, and all the study procedures were under the ethical standards of the Declaration of Helsinki. The study was divided into two phases: (1) cultural and linguistic adaptation and (2) instrument validity testing.

### 2.2. Cultural and Linguistic Adaptation to Brazilian Portuguese, Phase 1

The translation of the instrument into Portuguese was performed by independent translators (two native Brazilian Portuguese speakers and two native English speakers) who anonymously translated and later proofread the back-translation process results [18]. After completion, a different committee of five research group members reviewed and revised the translations and decided on the most appropriate [19,20].

The following step was the cultural adaptation. This phase aimed to ensure that foods and meals presented in the adapted version of the instrument were familiar to the target population and appeared as recognized dishes in Brazil. This process was performed following the first cultural adaptation of the tool [16]. Guidance for changing foods is relevant to the country; therefore, researchers used the Brazilian Dietary Guidelines [21] and information on food consumption in the nation reported by the Brazilian Family Budget Survey-Pesquisa de Orçamentos Familiares (POF) [22]. To determine changes, researchers had two criteria: (1) recognition of foods, packages, labels, and measurements, e.g., in the subscale of household food measurement, all measures originally reported in ounces were converted to grams, since Brazil uses the Internal System of Units; (2) nutritional context of the question, e.g., in the consumer skills subscale, the concept of “whole foods” compared with processed foods using fresh blueberries and blueberry juice was exchanged for grapes and grape juice, which was more relevant for the Brazilian population.

The research team used an agreement survey [23] involving six experts in nutrition (six registered dietitians from all five macroregional areas of Brazil: one each from the North, Northeast, Midwest, and South, and two from the Southeast—due to higher population density) to review the adapted instrument and provide feedback. The tool was sent to the experts remotely, and they individually ranked the adapted tool for relevance, wording, grammatical structure, and global readability on a 4-point scale. After experts ranked the tool, researchers reviewed the results, combined, and averaged the scores to finally test it by the Scale Content Validity Index (S-CVI). To calculate the global tool S-CVI, Item-S-CVI was averaged. An S-CVI value greater than 0.80 was considered acceptable [23]. All rankings and additional comments were considered for further modifications in the instrument.

Following the method used in the validation of Nlit with Spanish-Speaking Latinos in the US [16], after performing modifications based on experts’ review, the instrument was pilot-tested for comprehension and readability with a convenience sample of four patients from the target population of users of the Brazilian Public Health System—Sistema Unico de Saude (SUS). Cognitive interviews were used to evaluate the clarity of language and familiarity with food and meal items. Interviews engaged an open dialogue, collecting and discussing the participants’ opinions and interpretations as they answered the questions [16]. Interviews were audio-recorded and transcribed verbatim. We tested the tool readability through an adapted Fry Graph test, where a score greater than 60 was considered appropriate for the general adult population [24]. Participants’ views and readability testing helped to refine the familiarity and literacy level of the Brazilian Portuguese version of NLit.

An open discussion with the original NLit creator (Heather D. Gibbs, Ph.D., RD, LD) was carried out about the entire methodological approach to ensure that changes did not alter the intended NLit constructs.

### 2.3. Instrument Validity and Reliability, Phase 2

For validity testing, a convenience sample of *n* = 30 Brazilian adults, users from the Brazilian Public Health System, was recruited based on specific eligibility criteria, and the paper questionnaire containing the NLit-Br was administered. Recruitment was performed at a Brazilian Public Health System chronic disease clinic. One team member verified the inclusion and exclusion criteria for each subject interested in participating in the study based on their medical records. Inclusion criteria were the following: older than 18 years and diagnosed with at least one of the following chronic diseases: type 2 diabetes, hypertension, or chronic renal disease.

Potential study participants were informed about the study and then verbally consented. Each subject who verbally confirmed to participate was invited to fill out the questionnaires while waiting for their medical appointment. The researcher who consented the study participant was present during the study visit to answer any logistical questions. Questionnaires included the adapted version of the NLit-Br; and the short assessment of health literacy for adult Portuguese speakers (SAHLPA-18) to assess the general health literacy [25].

### 2.4. The Short Assessment of Health Literacy for Portuguese-Speaking Adults (SAHLPA-18)

The SAHLPA-18 is a questionnaire designed and used by researchers in Brazil and Portugal to measure health literacy [25,26]. In our research, the Portuguese version of the tool [25] was administered to evaluate similarities and differences between the construct of nutrition literacy and health literacy. The SAHLPA-18 is an 18-item questionnaire that is valid to assess Brazilian Portuguese speakers’ health literacy (r_s_ = 0.96; *p* < 0.0001 and ICC test–retest reliability of 0.91 (95% CI 0.76; 0.96). SAHLPA-18 takes about 3–6 min to be completed. A score between 0 and 14 suggests inadequate health literacy.

### 2.5. Sociodemographic Characteristics

The following sociodemographic characteristics were collected: age, sex, educational level, monthly household income, and occupational status. Data related to educational level, monthly household income, and race were collected by following the classification using the Health and Sociodemographic Characteristics in Brazil by the Brazilian Institute of Geography and Statistics (IBGE) [27].

### 2.6. Data Analysis

Data analysis was performed using IBM SPSS Statistics for Windows, version 22.0. Armonk, NY, USA: IBM Corp. For all of the analyses, a p-value equal to or less than 0.05 was considered significant. Data were presented as a percentage or mean and standard deviation (SD). Internal consistency of the entire instrument and its domains was evaluated by Kuder–Richardson Formula 20 (KR-20) [23,28]. Construct validity was determined by Spearman’s rho correlation between NLit-Br and SAHLPA-18.

## 3. Results

Approximately half of the sample was male (53.0%), with a mean age of 62, and the majority had an education level corresponding to less than high school (67%) (Table 1).

### 3.1. Phase 1

**Figure 1:**
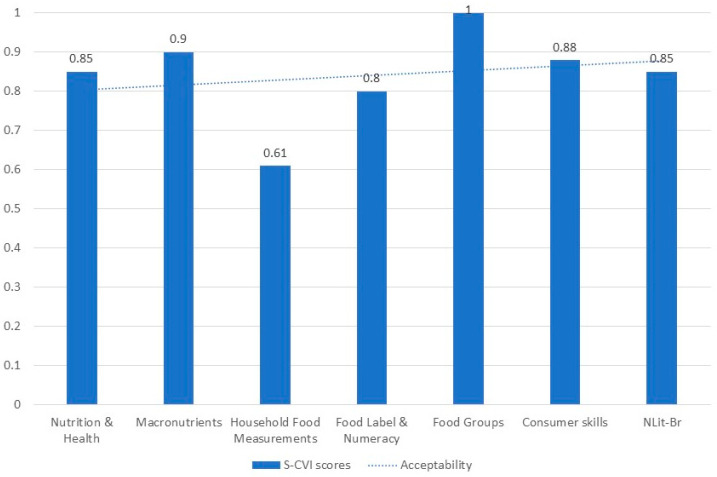
Nutrition experts’ agreement scores on the Nutrition Literacy Assessment Instrument in Brazilian Portuguese (*n* = 6).

Results from expert review indicated that overall NLit-Br was relevant and acceptable for Brazilians (S-CVI = 0.85). Experts scored all NLit-Br subscales with a score greater than 0.80, except for the subscale Household Food Measurement (S-CVI = 0.61) (Figure 1). Experts expressed that even though the subscale ‘Household Food Measurement’ is relevant in the context of weight management, participants with low education levels would have more difficulty comprehending it. Due to low S-CVI score from experts and in agreement with the author of the original instrument the subscale Household Food Measurement was excluded from the Brazilian version. The adapted NLit-Br had a readability score of 65 or 8th to 9th grade, which is considered acceptable.

Regarding the subscales of the instrument, the experts agree that adaptations needed to include food, meals, and images of products that were recognizable for Brazilians. In the first subscale, “Nutrition and Health,” all foods were reviewed, and changes were performed to the Brazilian context, maintaining the same macronutrient content.

In the second subscale, “Energy Sources in Food” foods and meals were adapted to the Brazilian context, and measurements were adapted to the international metric system. In the subscale “Food Label and Numeracy,” researchers used a Brazilian food label with similar nutrition content. Researchers maintained the groups in the “Food Groups” subscales groups and adapted foods for the Brazilian context. In the last subscale, “Consumer skills,” researchers adapted all foods and pictures to the Brazilian context and maintained the content of the subscale (Table 2).

### 3.2. Phase 2

The NLit was validated in Brazilian-Portuguese (NLit-Br—Supplementary file). The mean scores for SAHLPA-18 and NLit-Br were 12 (SD 4.5) and 29 (SD 8.3), respectively. The reliability of NLit-Br was good (KR-20 = 0.868). The NLit-Br and SAHLPA-18 scores were positively correlated (r = 0.665, *p* < 0.001). Four out of five NLit-Br subscales were positively correlated with SAHLPA-18 (Table 3).

## 4. Discussion

In the present study, the reliability and validity of the translated and adapted questionnaire for assessing nutrition literacy in the Brazilian context (NLit-Br) were assessed. Results from this study demonstrated the validity (r = 0.665, *p* < 0.001) and reliability (KR-20 = 0.868) of NLit-Br.

The NLit-Br achieved a slightly lower agreement than the validation study of NLit to Spanish (NLit-S) [16]. In the Brazilian version, the lower score resulted from the experts’ evaluation of the subscale Household Food Measurements, which was considered a subscale of difficult interpretation. Similar to the conclusion of the nutrition experts, the current Brazilian Dietary Guidelines also does not include the concept of food portion with the premise that food can be combined in a wide variety of proportions (e.g., culinary preparations) that would be hard to be self-reported [21]. Future studies on nutrition literacy should explore innovative strategies to measure the concept of food portions among Brazilian population considering the concept may be difficult to understand, but it does not exclude its crucial role in healthy eating, prevention, and management of obesity [29,30].

According to the group of experts’ considerations, the other items that required adaptations were directly related to cultural relevance. The adaptations of the “Nutrition and Health” domain, for example, sought to meet cultural characteristics that directly affect the perception of food as something healthy or not. In a study by Gomez and Torelli [31], the authors identified how specific appeals for nutrition information could arouse different reactions depending on the reader’s culture. Therefore, evaluating images and information about what is healthy or not is directly related to experiences and information that make up the food image for the target audience and justifies the changes made.

On the other hand, the decision of the group of experts on the maintenance of information on macronutrients observes well-established practical and scientific aspects concerning the topic [29,30]. For food selection to make a healthy meal, understanding its composition is one tool that can make a difference. Conscious eating comprises elements that range from nutritional composition to food preparation. The term ‘food literacy’ is widely used by studies that aim to describe the proficiency level to access and understand food information and skills and abilities to apply the obtained food information [32]. However, few food literacy studies incorporate how the concept interacts with health outcomes [32], which is an essential factor when working with people affected by nutrition-related chronic diseases that must know what to eat and when to eat a particular food. In this way, the person is empowered to plan their meals based on all the personal and environmental aspects that can lead to food choices [33].

Results from the correlation between SAHLPA and NLit-Br showed that both instruments measured the same global concept of literacy. A similar correlation was found by Gibbs et al. in the first cross-cultural adaptation of the instrument to Spanish [16], but not with the Italian version [17]. The Brazilian adaptation had a higher reliability score for the entire NLit-Br instrument than the single subscale, similar to the adapted NLit tool to Italian (NLit-IT) [17]. The higher reliability for the NLit-Br as a whole suggests the administration of the entire instrument instead of a single subscale to assess the construct of nutrition literacy. Different from the English and Italian versions [15,17], but similar to the Spanish version [16], the NLit-Br also did not measure food intake or explore if NLit is a predictor of diet quality.

The reported low scores on health literacy and nutrition literacy for the sample included in this study raises questions about the importance of age and education level for nutrition literacy. The present sample was elderly with low education levels. Previous studies in nutrition literacy obtained higher scores and had a wide age range of adults [14,16,17,34]. One study of 775 older adults with a mean age 81 years found that higher health literacy was associated with better cognitive health and decreased incidence of Alzheimer’s [35]. Likewise, an analysis of health literacy data collected across 8 European countries found that older age was associated with poorer health literacy across countries [36].

In Brazil specifically, a previous study assessed nutrition literacy in a sample of young adults using the Nutritional Literacy Scale [37], but it is difficult to draw conclusions about nutrition literacy from the two studies due to differences in measures and sample characteristics. In that study, the scores for nutrition literacy were higher, and most achieved a good nutrition literacy level compared with the present study. However, in addition to their younger age, the sample also had a higher education level, both of which are factors that could mediate or moderate nutrition literacy [37].

Brazil’s population aging faster than any other country worldwide. Brazilian census predicted that, by 2040, the population would comprise 153 elderly people for every 100 children. Miranda et al. reported that the country is not ready for this demographic change, noting that Brazil’s significant social inequalities for the elderly reflect higher morbidity, disability, and lack of access to quality health care [38]. Data from ELSI-Brazil, an aging cohort with more than 9000 participants, found an inverse association amongst the elderly where lower education was associated with higher functional disability [39]. Another cohort conducted with the elderly population in a small rural town in Minas Gerais found elderly with low income and low education presented environmental risk behaviors, including lower consumption of fruits and vegetables [40].

This study has some limitations. First, it was subjected to common survey limitations such as self-reported data and social desirability. Future studies that are larger and representative of diverse regions and demographics are needed to robustly validate the NLit-Br. Second, there was no test–retest to demonstrate the reproducibility of the instrument. However, this study accomplished an important step of cross-cultural adaptation and validation of the tool and included nutrition experts representing all regions across the country of Brazil. Lastly, food consumption habits were not collected, which would be useful to evaluate for convergent validity and establish the cut-off points for low, medium or high NL, given the English and Italian versions.

## 5. Conclusions

Nutrition is crucial for promoting health and managing chronic diseases that are leading causes of mortality in Brazil. The NLit-Br emerged as a tool that would support personalizing nutrition education and nutrition policies for Brazil. The NLit-Br is the first of its kind to be translated to Brazilian Portuguese and be culturally adapted to Brazil. The NLit-Br is a reliable tool to measure nutrition literacy in Brazil, incorporating Brazilian food culture, Brazilian diet guidelines, and nutrition professionals’ expertise for the entire country. While the NLit-Br is a reliable instrument for measuring the nutrition literacy of Brazilians, more research is needed to understand the relationship between nutrition literacy and dietary behavior for Brazilians.

## Figures and Tables

**Table 1:** Sociodemographic, health literacy and nutrition literacy data for Brazilians (*n* = 30).

	*n*	Mean (Standard Deviation) or [%]
Sociodemographic characteristics		
Age at enrollment, years	30	62.0 (9.9)
Sex		
Female	14	(47.0)
Male	16	(53.0)
Race		
White	13	(43.0)
Brown	8	(27.0)
Black	9	(30.0)
Monthly Household Income		
1 to 2 minimum wage ^a^	17	[56.7]
2 to 3 minimum wage ^a^	7	[23.3]
More than 3 minimum wage ^a^	6	[20.0]
Education		
Less than high school	20	[67.0]
High school graduate	6	[20.0]
Some college or more	4	[13.0]
Occupational status		
Employed	10	[33.0]
Retired	20	[67.0]
Health & Nutrition Literacy		
SAHLPA-18 score	30	11 (8.2)
NLit-Br score	30	36.0 (8.5)

^a^ Brazilian minimum wage: BRL 880/USD 295.

**Table 2:** Example of cultural adaptation and substitution of the original instrument items for Brazilian context.

Subscale	NLit	NLit-Br
Nutrition and health	An example of a food with added sugars is: a. Milk b. Baby carrots c. Brown rice d. Chocolate pudding	Um exemplo de alimento com adição de açúcar é o (a): a. Leite b. Cenoura c. Arroz integral d. Pudim de leite condensado
Energy sources in food	Which breakfast is highest in carbohydrate? a. 8 oz. orange juice, 2 slices of toast with strawberry jam b. 8 oz. orange juice, 2 scrambled eggs c. 8 oz. reduced-fat milk, 2 slices of toast with peanut butter d. 8 oz. reduced fat-milk, 2 slices of bacon	Qual café da manhã é o que contem mais carboidratos? a. 240 mL de suco de laranja, 2 fatias de torrada com geleia de morango b. 240 mL de suco de laranja, 3 fatias de queijo tipo minas c. 240 mL leite semidesnatado, 2 fatias de torrada com manteiga d. 240 mL de leite semidesnatado, 2 fatias de presunto cozido
Food label and numeracy	If you are limiting your total fat intake for 65 g per day, and you eat one (1) cup of macaroni and cheese, what is the highest of total fat you can eat from other food sources? a. 33 g b. 47 g c. 53 g d. 57 g	Se você está limitando o consume total de gordura para 65 gramas por dia, e você come um (1) pacote de macarrão instantâneo. Olhando a quantidade de gordura no rótulo, qual é o total de gordura que você pode comer de outros alimentos? a. 39 g b. 16 g c. 49 g d. 59 g
Food groups	This is a list of foods. Using the chart below, write the name of each food in the food groups in which it belongs according to its nutrition value. a. Apple b. Milk c. Flour tortilla d. Regular salad dressing	Esta é uma lista de alimentos. Usando o Quadro abaixo, escreva o nome de cada alimento no seu grupo pertencente, de acordo com o seu valor nutricional. a. Maçã b. Leite c. Aveia d. Maionese
Consumer skills	If calories are equal for one serving of each food, which provides the most healthful nutrients overall? a. Applesauce with no sugar added b. Apple c. Applesauce with no sugar added is equal to an apple in nutrition 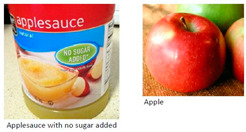	Se as calorias são iguais para uma porção de cada alimento, qual alimento tem mais nutrientes? a. Suco de abacaxi industrializado sem adição de açúcar b. Abacaxi c. Suco de abacaxi industrializado sem adição de açúcar tem a mesma quantidade de nutrientes do que o abacaxi 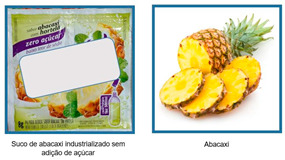

**Table 3:** Validity and reliability statistics for the Nutrition Literacy Assessment Instrument in Brazilian Portuguese (*n* = 30).

	Number of Items	Correlation with SAHLPA-18 (Spearman’s ᵣ)	Internal Consistency (KR-20)
Nutrition and Health	10	0.594 **	0.525 ′
Energy sources in food	10	0.576 **	0.299 ′
Food Label and Numeracy	10	0.449 *	0.520 ′
Food Groups	16	0.534 **	0.817 ′′′
Consumer Skills	9	0.361	0.474 ′
NLit-Br	55	0.665 **	0.868 ′′′

NLit-Br = Nutrition Literacy Assessment Instrument—Brazilian Portuguese; SAHLPA-18 = Short Assessment of Health Literacy-Portuguese; *p* = < 0.05 *; *p* < 0.01 **; We classified KR-20 as follows: <0.70 is low ′, 0.80–0.89 is good ′′′.

## Data Availability

Data from this manuscript is part of L.B.d.S.S dissertation Instrumento de Avaliação do Letramento em Nutrição para a População Brasileira: Adaptação Transcultural.

## Supplementary Materials

The following supporting information can be downloaded at: https://www.mdpi.com/article/10.3390/nu14224914/s1, Nutrition Literacy Assessment Instrument for Brazilians (NLit-Br).
