# Supplementary material for: The Nutrition Literacy Assessment Instrument for Brazilians, NLit-Br: An Exploratory Cross-Cultural Validity Study
Source: Nutrients. 2022 Nov 20;14(22):4914. doi: 10.3390/nu14224914 (PMC9693279; doi:10.3390/nu14224914)
Supplement: Supplementary file 1 [file nutrients-14-04914-s001.zip › nutrients-2038766-supplementary.pdf]

**Instrumento de Avaliação de Letramento Nutricional (NLit)**

Código do participante: _____________

Início do preenchimento: ____________

Fim do preenchimento: _____________

Essa ferramenta irá ajudar a equipe de pesquisa a ter uma ideia das informações sobre nutrição que você já sabe e o que pode ser novo para você.

****Por favor, continue na próxima página****

**Nutrição e Saúde**

**Orientações:** Por favor, leia o texto abaixo e responda as seguintes questões.

Manter uma alimentação saudável e se exercitar são fatores-chave para uma boa saúde e qualidade de vida. Uma dieta saudável ajuda a prevenir, retardar ou melhorar as doenças crônicas. Algumas dessas doenças incluem: doenças do coração, hipertensão, diabetes tipo 2 e câncer. Uma alimentação saudável também ajuda as pessoas a atingir um peso adequado.

Uma alimentação saudável tem grande quantidade de **alimentos ricos em nutrientes**, como frutas, legumes e grãos integrais. Uma alimentação saudável também tem uma baixa quantidade de **alimentos ricos em calorias**, como farinhas refinadas e alimentos com adição de açúcar. Apesar desses alimentos proverem energia, muita energia pode levar a ganho de peso e doenças crônicas.

Para seguir uma alimentação saudável, **coma mais**:

- **Alimentos ricos em nutrientes:** Frutas e vegetais são excelentes fontes de vitaminas e minerais, além de outros nutrientes necessários para a saúde. Ao mesmo tempo, eles tem uma baixa quantidade de calorias. Comer mais desses alimentos pode ajudar no controle de peso e a diminuir o risco de doenças.
- **Alimentos integrais:** Alimentos feitos de sementes inteiras de trigo, arroz, aveia ou cevada são conhecidos como alimentos integrais. Alimentos integrais são ricos em nutrientes e podem promover controle de peso e diminuição do risco de doenças.
- **Alimentos ricos em proteínas com baixo teor de gordura:** Alimentos como peixe, frango e peru (sem pele), laticínios com baixo teor de gordura, carne magra, feijão e lentilha, são também alimentos ricos em nutrientes quando preparados com poucas calorias adicionadas.

Para seguir uma alimentação saudável, **coma menos**:

- **Alimentos ricos em calorias:** Calorias são fontes de energia para o corpo. Alguns alimentos, como batatas chips, refrigerante, açúcar, sucos de frutas industrializados e sobremesas são ricos em calorias e pobres em nutrientes necessários. Esses alimentos são conhecidos como alimentos ricos em calorias. Comer muito esses alimentos pode levar a um aumento de peso e do risco de doenças.
- **Alimentos refinados:** Esses alimentos perdem parte do grão que é rico em nutrientes. Alguns exemplos incluem o pão branco, biscoitos em geral, o arroz branco e o macarrão. Alimentos refinados são pobres em nutrientes necessários e muitos podem levar ao ganho de peso.
- **Gordura saturada** e **colesterol** são nutrientes principalmente encontrados em alimentos de origem animal, como carnes gordas e laticínios integrais. Esses

nutrientes podem aumentar os níveis de colesterol no sangue, uma condição que pode reduzir o fluxo de sangue para o coração.

- **Sódio** é encontrado no sal de cozinha e em vários alimentos industrializados. Ingerir menos sódio pode diminuir a pressão arterial. A pressão alta é prejudicial, porque pode fazer com que o coração tenha que trabalhar mais.
- **Alimentos com adição de açúcar** são alimentos que tem altos níveis de açúcar e poucos nutrientes, como refrigerantes, sobremesas e doces. Comer muito desses alimentos pode levar ao ganho de peso e causar aumento do açúcar no sangue para aqueles que tem diabetes.

****Por favor, vá para a próxima página para responder as questões sobre o texto****

**Instruções:** Escolha a melhor resposta para as questões abaixo. Você pode voltar a ler o texto da página anterior para ajudar a escolher suas respostas.

1. Alimentos como __________ devem ser incluídos com maior frequência em uma dieta saudável.

   A. carne vermelha
   B. manteiga
   C. cereais integrais
   D. farinhas refinadas

2. Um exemplo de alimento rico em calorias é o (a) __________.

   A. sorvete
   B. aveia
   C. laranja
   D. cenoura

3. Alimentos com muitos nutrientes como os (as) __________ deve ser consumida (o) mais frequentemente.

   A. refrigerantes
   B. batatas fritas
   C. frutas
   D. sucos industrializados

4. A couve-flor é um exemplo de alimento que é rico em__________.

   A. energia
   B. proteína
   C. nutrientes
   D. calorias

5. A Gordura saturada é mais encontrada em alimentos__________.

   A. de origem animal
   B. de origem vegetal
   C. fontes de grãos
   D. fontes de açúcar

6. Qual refeição tem mais nutrientes?

   A. Hambúrguer com pão, batatas chips, leite desnatado
   B. Macarrão com molho de carne, pão de alho, leite desnatado.
   C. Frango sem pele, feijão cozido no vapor, fruta
   D. Costeleta de porco, arroz branco cozido no vapor, leite desnatado.

7. Diminuir a ingestão de sal na alimentação pode diminuir o (a) __________, sendo bom para o coração.

A. glicose sanguínea
B. pressão arterial
C. sódio no sangue
D. colesterol no sangue

8. Um exemplo de alimento com adição de açúcar é o (a) __________.

A. leite
B. cenoura
C. arroz integral
D. pudim de leite condensado

9. Um exemplo de bebida com muitas calorias é o __________.

A. refrigerante diet
B. suco industrializado
C. café preto
D. chá sem açúcar

10. Qual dos seguintes alimentos tem mais sódio?

A. Catchup
B. Brócolis congelado
C. Suco de laranja natural
D. Morangos

**Macronutrientes:** Essas questões são sobre carboidratos, proteínas e gorduras, que são fontes de energia para o corpo.

**Instruções:** Use os seus conhecimentos sobre nutrição para responder as perguntas abaixo.

11. As calorias dos alimentos como azeite e manteiga vem do seu alto conteúdo de __________.

A. vitamina
B. carboidrato
C. proteína
D. gordura

12. O __________ encontrado no suco de laranja natural é um tipo de carboidrato.

A. açúcar
B. cálcio
C. amido
D. folato

13. Uma boa fonte de __________ é encontrada em alimentos como ovos, frango e peixe.

A. amido
B. proteína
C. fibra
D. carboidrato

14. Qual grupo de alimentos fornece mais proteína?

A. Pão, arroz, macarrão.
B. Banana, vinagre, brócolis.
C. Costeleta de porco, ovo, queijo.
D. Coalhada, azeite de oliva, molho para salada.

15. Qual grupo de alimentos fornece mais carboidratos?

A. Aveia, arroz, batata.
B. Ovo, manteiga, queijo.
C. Amendoim, bacon, óleo vegetal.
D. Frango, linguiça, peixe.

16. Qual grupo de alimentos fornece mais gordura?

A. Arroz, tapioca, biscoito de sal.
B. Batata, pera, leite.
C. Cenoura, abacate, iogurte.
D. Maionese, margarina, azeite de oliva.

17. Qual café da manhã é o que contem mais carboidratos?

A. Suco de laranja, torrada com geleia de morango
B. Suco de laranja, queijo
C. Leite semidesnatado, torrada com manteiga
D. Leite semidesnatado, presunto

18. Se o seu médico fala para você comer mais proteína, qual alimento é a melhor escolha para aumentar a proteína e gorduras saudáveis em sua alimentação?

A. Peixe
B. Linguiça
C. Queijo
D. Pão

19. Se o seu médico te fala para você comer menos gordura, qual alimento você deve comer com menor frequência?

A. Feijão preto
B. Maionese
C. Batata
D. Pão

20. O Azeite de oliva é mais saudável que a margarina porque:

A. é natural
B. tem menos gordura
C. tem menos calorias
D. tem tipos de gordura mais saudáveis.

**Rótulo de alimentos e Números**

Este rótulo de alimento à direita foi retirado de um pacote de macarrão instantâneo.

Embalagem de macarrão instantâneo

| INFORMAÇÃO NUTRICIONAL<br>Porção de 85g (1 pacote)<br>80g de macarrão + 5g de tempero | | |
|---|---|---|
| | **Quantidade por porção** | **%VD (*)** |
| **Valor energético** | 388 kcal = 1629 kJ | 19% |
| **Carboidratos** | 54 g | 18% |
| **Proteínas** | 7,0 g | 9% |
| **Gorduras totais** | 16 g | 29% |
| **Gorduras saturadas** | 7,6 g | 34% |
| **Gorduras *trans*** | 0 g | **** |
| **Fibra alimentar** | 1,0 g | 4% |
| **Sódio** | 1630 mg | 68% |

*** % Valores Diários de referência com base em uma dieta de 2000 kcal ou 8400 kJ. Seus valores diários podem ser maiores ou menores dependendo de suas necessidades energéticas.**
**** VD não estabelecido.**

21. Quantas calorias você irá consumir se comer **todo o pacote**?
   A. 259 calorias
   B. 388 calorias
   C. 184 calorias
   D. 738 calorias

22. Se você está tentando comer menos de 500mg de sódio por refeição, quanto desse alimento você pode comer, sem nenhum acompanhamento?
   A. Menos da metade do pacote
   B. Metade do pacote
   C. Mais da metade do pacote
   D. O pacote inteiro

23. Se você está limitando o consumo total de gordura para 65 gramas por dia, e você come um (1) pacote de macarrão instantâneo. Olhando a quantidade de gordura no rótulo, qual é o total de gordura que você pode comer de outros alimentos?
   A. 39 gramas
   B. 16 gramas
   C. 49 gramas
   D. 59 gramas

24. Qual é o total de carboidrato em gramas que você comeria em meio pacote de macarrão instantâneo?
   A. 15 gramas
   B. 25 gramas
   C. 27 gramas
   D. 54 gramas

25. Se você é aconselhado a aumentar sua ingestão de fibras, o macarrão é uma boa escolha alimentar?
   A. Sim
   B. Não

26. Se você está tentando limitar sua ingestão de gorduras saturadas para 7 gramas por refeição, quanto de macarrão instantâneo você pode comer, sem acompanhamento?

A. O pacote inteiro
B. Menos de um pacote inteiro
C. Metade do pacote
D. Mais da metade do pacote

27. Se você comer meio pacote deste macarrão instantâneo, qual o total de gordura em gramas que você irá comer?

A. 7 gramas
B. 16 gramas
C. 8 gramas
D. 6 gramas

Embalagem de macarrão instantâneo

| INFORMAÇÃO NUTRICIONAL<br>Porção de 85g (1 pacote)<br>80g de macarrão + 5g de tempero | | |
|---|---|---|
| | **Quantidade por porção** | **%VD (*)** |
| **Valor energético** | 388 kcal = 1629 kJ | 19% |
| **Carboidratos** | 54 g | 18% |
| **Proteínas** | 7,0 g | 9% |
| **Gorduras totais** | 16 g | 29% |
| **Gorduras saturadas** | 7,6 g | 34% |
| **Gorduras *trans*** | 0 g | **** |
| **Fibra alimentar** | 1,0 g | 4% |
| **Sódio** | 1630 mg | 68% |

*** % Valores Diários de referência com base em uma dieta de 2000 kcal ou 8400 kJ. Seus valores diários podem ser maiores ou menores dependendo de suas necessidades energéticas.**
**** VD não estabelecido.**

28. Quantos gramas de proteína você irá consumir se comer um (1) pacote?

A. 7,6 gramas
B. 7,0 gramas
C. 3,5 gramas
D. 1,0 gramas

29. Se você é aconselhado a comer 45 gramas de carboidrato **por refeição**, e come meio pacote de macarrão instantâneo, qual a quantidade de carboidratos em gramas que você deve comer de outro alimento na mesma refeição?

A. 9 gramas
B. 10 gramas
C. 18 gramas
D. 20 gramas

30. Se seu médico te aconselha a limitar o consumo total de gordura para 64 gramas **por dia**, qual a porcentagem diária de gordura que você terá consumido se comer um pacote de macarrão instantâneo?

A. 10%
B. 15%
C. 18%
D. 25%

**Grupos alimentares** **Código: _________**

Esta é uma lista de alimentos. Usando o quadro abaixo, escreva o nome de cada alimento no seu grupo pertencente, de acordo com o seu valor nutricional. Exemplo: ***Pão*** (veja abaixo)

| | | | |
|---|---|---|---|
| 31. Maçã | 35. Queijo | 39. Costela de porco | 43. Alface |
| 32. Leite | 36. Arroz branco | 40. Cenoura | 44. Banana |
| 33. Macarrão | 37. Aveia | 41. Manteiga | 45. Maionese |
| 34. Suco de Laranja Natural | 38. Frango | 42. Suco industrializado | 46. Geleia de Frutas |

| **Grãos** | **Vegetais** | **Frutas** | **Proteína** | **Laticínios** | **Óleos e gorduras** | **Açucares adicionados** |
|---|---|---|---|---|---|---|
| ***Pão*** | | | | | | |

**Habilidades como consumidor**

**Instruções:** Escolha a melhor resposta para as questões a seguir.

47. Se as calorias são iguais para uma porção de cada alimento, qual alimento tem mais nutrientes?

A. Suco de abacaxi industrializado sem adição de açúcar
B. Abacaxi
C. Suco de abacaxi industrializado sem adição de açúcar tem a mesma quantidade de nutrientes do que o abacaxi.

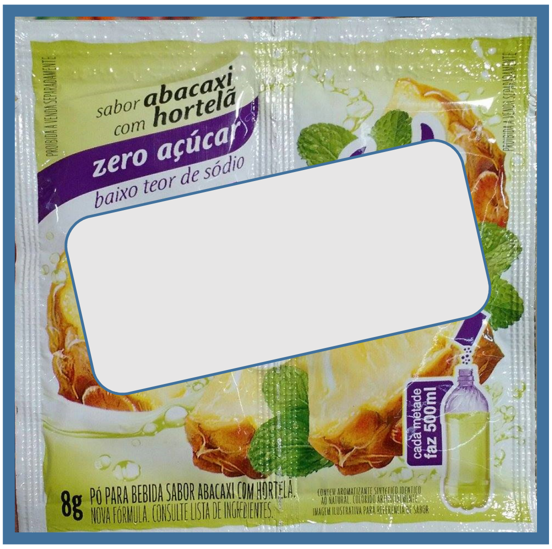


Suco de abacaxi industrializado sem adição de açúcar

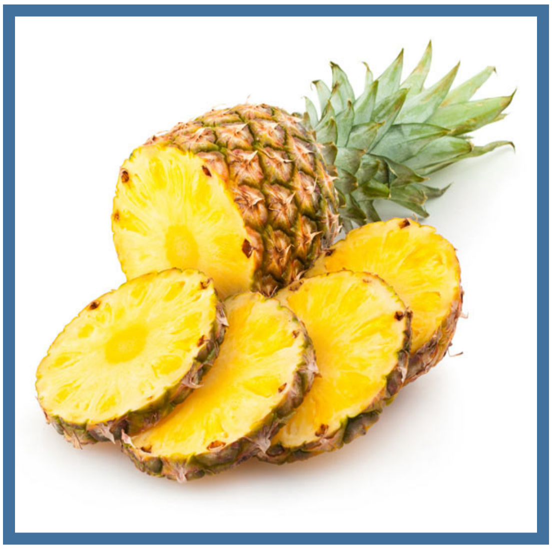

Abacaxi

48. Se as calorias são iguais para uma porção de cada alimento, qual alimento é o lanche mais saudável?

A. Barra de fruta
B. Uva passa
C. Barra de fruta é igual a uva passa em termos nutricionais

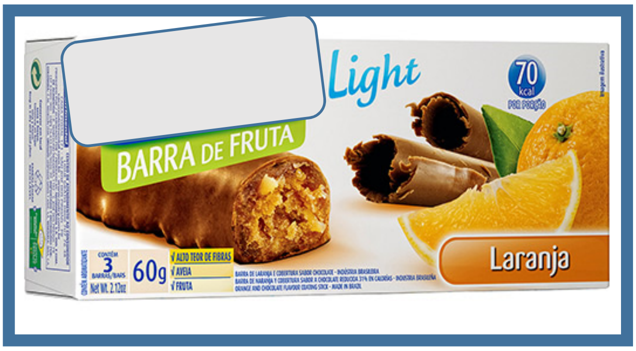


Barra de fruta

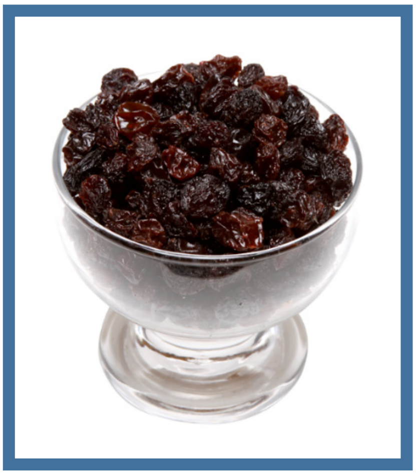

Uva passa

**Instruções:** Escolha a melhor resposta para as questões a seguir.

49. Se as porções são iguais, qual carne tem menos gordura?

A. Contrafilé

B. Patinho

C. Contrafilé e Patinho tem a mesma quantidade de gordura.

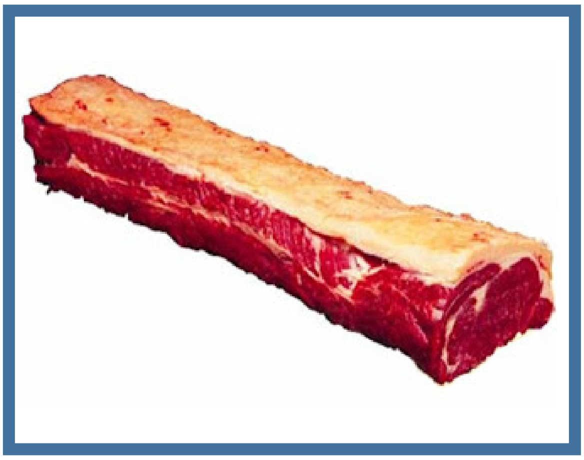

Contrafilé

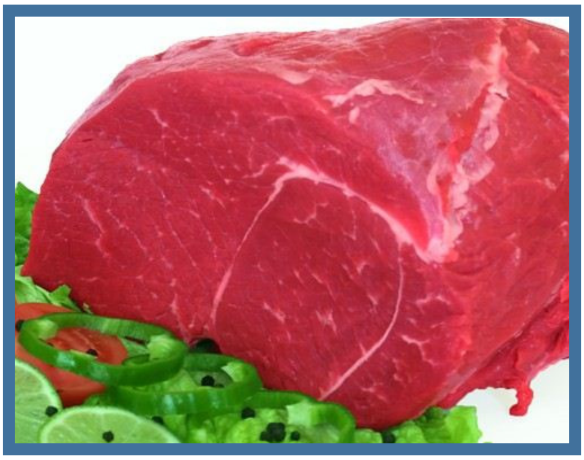

Patinho

50. Qual bebida tem mais calorias por copo (240mL)?

A. Leite desnatado

B. Suco de goiaba industrializado

C. Leite desnatado e suco de goiaba industrializado são iguais em calorias

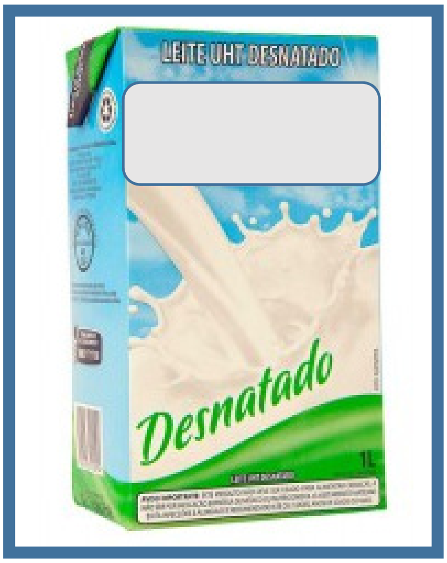


Leite desnatado

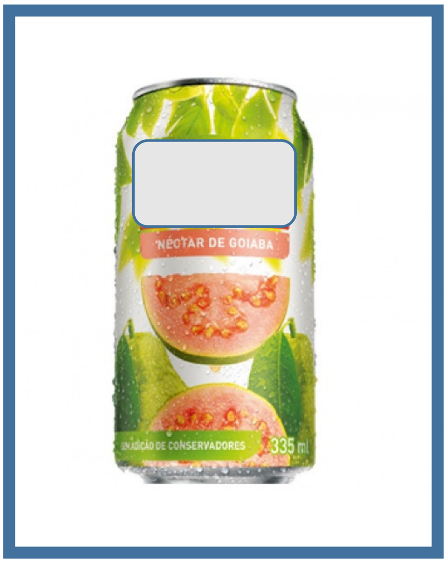


Suco de goiaba industrializado

51. Qual opção de ervilha tem menos sódio?

A. Ervilha enlatada

B. Ervilha seca

C. Ervilha enlatada e ervilha seca tem a mesma quantidade de sódio

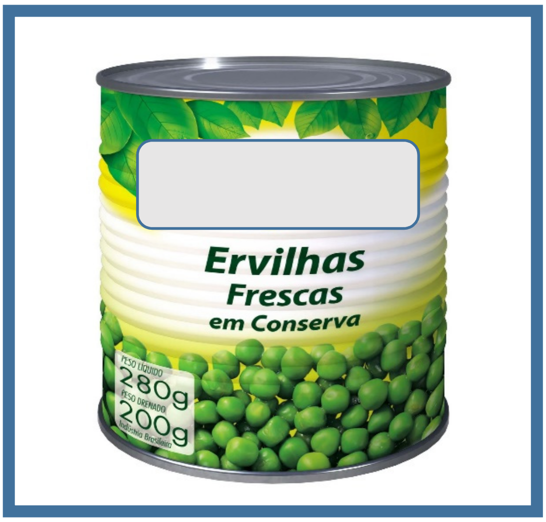


Ervilha enlatada

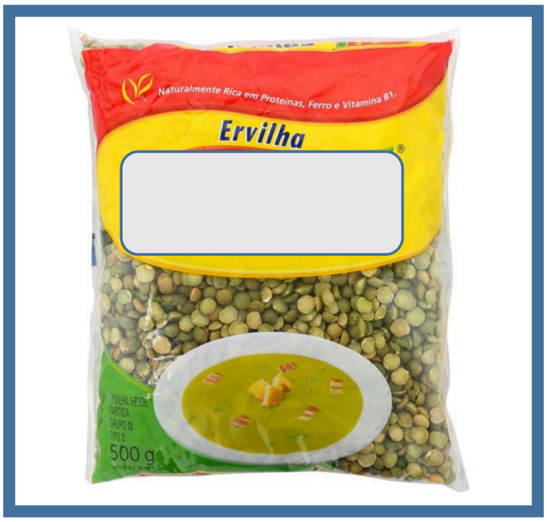


Ervilha seca

52. Qual tipo de salada verde tem mais nutrientes?

A. Alface

B. Couve

C. Alface e couve tem a mesma quantidade de nutrientes.

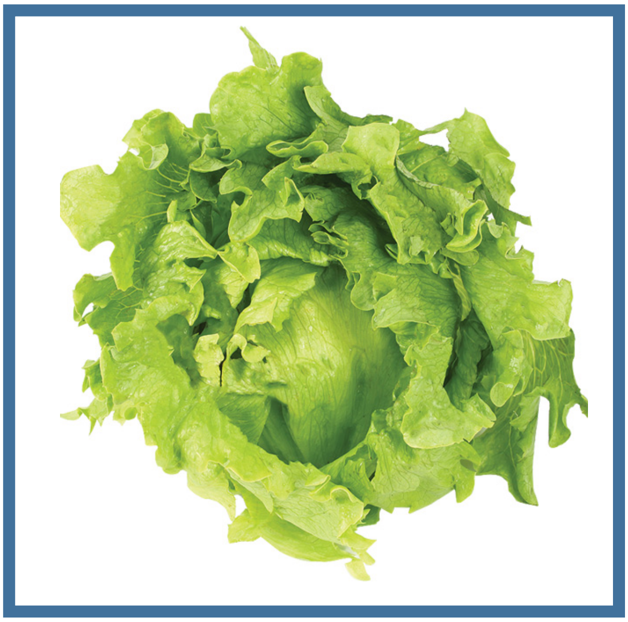

Alface

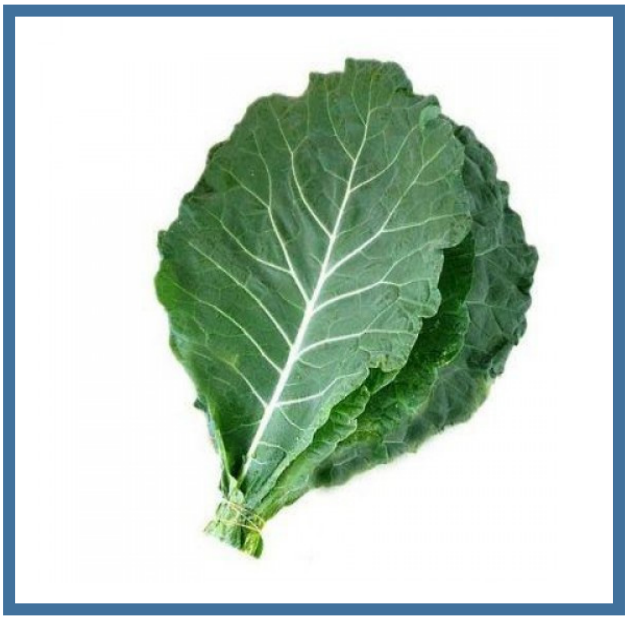

Couve

53. Qual parte do rótulo de alimento fornece a melhor informação sobre a quantidade de açúcar?

A. Informação nutricional
B. Informação da embalagem que diz “Diet”
C. A informação nutricional e a informação da embalagem que diz “Diet” são fontes iguais de informação sobre a quantidade de açúcar.

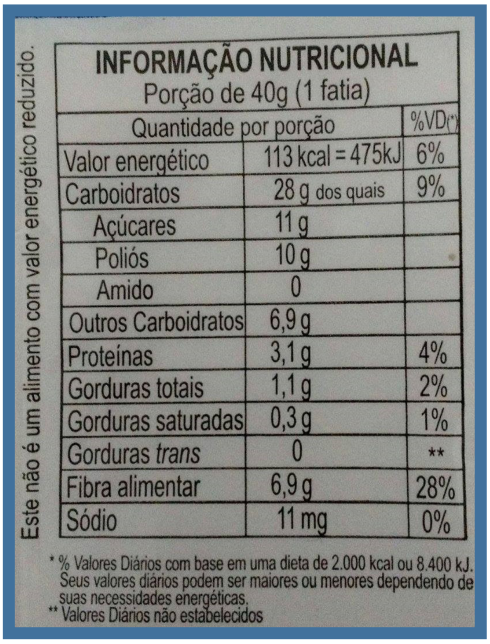

Este não é um alimento com valor energético reduzido.

**INFORMAÇÃO NUTRICIONAL**
Porção de 40g (1 fatia)

| Quantidade por porção | | %VD(*) |
|---|---|---|
| Valor energético | 113 kcal = 475kJ | 6% |
| Carboidratos | 28 g dos quais | 9% |
| Açúcares | 11 g | |
| Poliós | 10 g | |
| Amido | 0 | |
| Outros Carboidratos | 6,9 g | |
| Proteínas | 3,1 g | 4% |
| Gorduras totais | 1,1 g | 2% |
| Gorduras saturadas | 0,3 g | 1% |
| Gorduras *trans* | 0 | ** |
| Fibra alimentar | 6,9 g | 28% |
| Sódio | 11 mg | 0% |

* % Valores Diários com base em uma dieta de 2.000 kcal ou 8.400 kJ. Seus valores diários podem ser maiores ou menores dependendo de suas necessidades energéticas.
** Valores Diários não estabelecidos

Informação nutricional

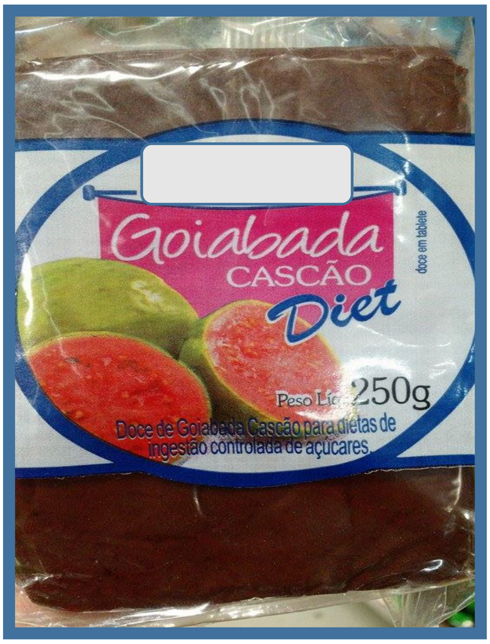


Informação do pacote que diz “Diet”

54. Se as calorias são iguais, qual alimento é mais nutritivo?

A. Uva
B. Suco de uva
C. Uva e suco de uva tem a mesma quantidade de nutrientes

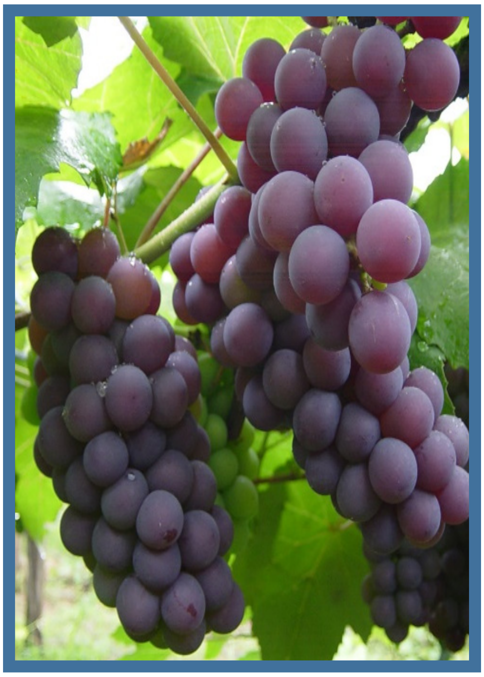

Uvas

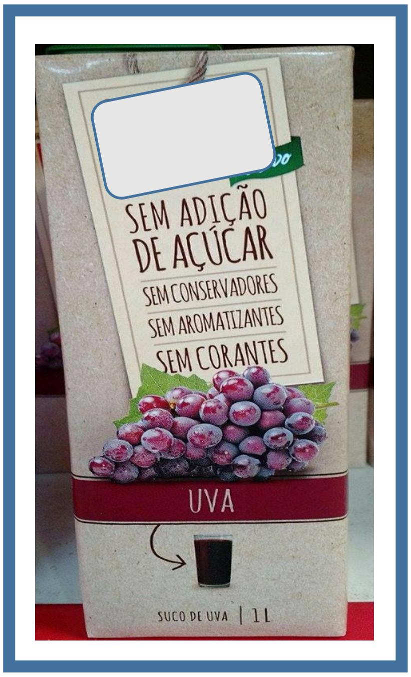


Suco de uva

55. Qual parte do rótulo de alimentos tem a melhor informação para a escolha de um alimento integral?

A. Pacote com a afirmação "Integral"
B. Lista de ingredientes
C. Pacote com a afirmação "Integral" e lista de ingredientes são fontes iguais de informação nutricional.

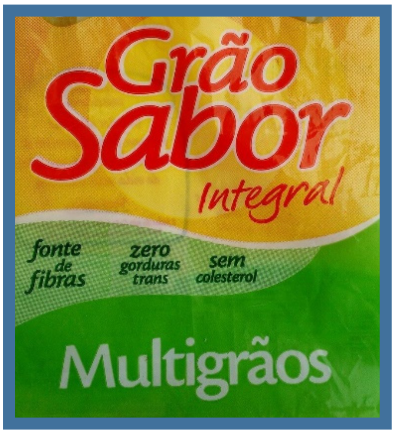


Pacote com a afirmação "Integral"

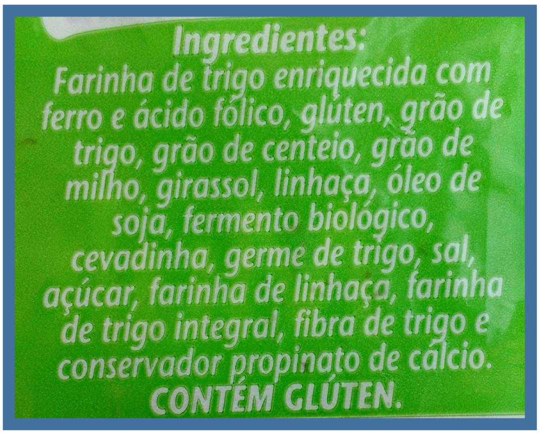


Lista de ingredientes

**PARE AQUI. Por favor, entregue esse instrumento para a equipe de pesquisa.**
